# Effectiveness of the fun for wellness online behavioral intervention to promote well-being and physical activity: protocol for a randomized controlled trial

**DOI:** 10.1186/s12889-019-7089-2

**Published:** 2019-06-13

**Authors:** Nicholas D. Myers, Isaac Prilleltensky, Seungmin Lee, Samantha Dietz, Ora Prilleltensky, Adam McMahon, Karin A. Pfeiffer, Morgan E. Ellithorpe, Ahnalee M. Brincks

**Affiliations:** 1 Department of Kinesiology, Michigan State University, 201 IM Sports Circle Building, 308 W. Circle Drive, East Lansing, MI 48824 USA; 2 School of Education and Human Development, University of Miami, Miami, USA; 3 Department of Advertising and Public Relations, Michigan State University, East Lansing, USA; 4 Department of Epidemiology and Biostatistics, Michigan State University, East Lansing, USA

**Keywords:** E-health, M-health, Self-efficacy theory

## Abstract

**Background:**

Fun For Wellness (FFW) is an online behavioral intervention developed to encourage growth in well-being by providing capability-enhancing learning opportunities to participants. Self-efficacy theory guides the conceptual model underlying the FFW intervention. Some initial evidence has been provided for the efficacy of FFW to promote: well-being self-efficacy; interpersonal, community, psychological and economic subjective well-being; and, interpersonal and physical well-being actions. The purpose of this paper is to describe the protocol for a new randomized controlled trial (RCT) designed to provide the first investigation of the effectiveness of FFW to increase well-being and physical activity in adults with obesity in the United States of America.

**Methods:**

The study design is a large-scale, prospective, parallel group RCT. Approximately 9 hundred participants will be randomly assigned to the FFW or Usual Care (UC) group to achieve a 1:1 group (i.e., FFW:UC) assignment. Participants will be recruited through an online panel recruitment company. Data collection, including determination of eligibility, will be conducted online and enrollment is scheduled to begin on 8 August 2018. Data collection will occur at baseline, 30 days and 60 days after baseline. Instruments to measure demographic information, anthropometric characteristics, self-efficacy, physical activity and well-being will be included in the battery. Data will be modeled under an intent to treat approach and/or a complier average causal effect approach depending on the level of observed engagement with the intervention.

**Discussion:**

The effectiveness trial described in this paper builds upon the 2015 FFW efficacy trial and has the potential to be important for at least three reasons. The first reason is based upon a general scientific approach that the potential utility of interventions should be evaluated under both ideal (e.g., more controlled) and real-world (e.g., less controlled) conditions. The second reason is based upon the global need for readily scalable online behavioral interventions that effectively promote physical activity in adults. The third reason is based upon the troubling global trend toward obesity along with evidence for obesity as a risk factor for several major non-communicable diseases.

**Trial registration:**

ClinicalTrials.gov*, identifier: **NCT03194854**, registered 21 June 2017.*

**Electronic supplementary material:**

The online version of this article (10.1186/s12889-019-7089-2) contains supplementary material, which is available to authorized users.

## Background

The purpose of this protocol paper is to describe an RCT designed to provide the first investigation of the effectiveness of the Fun For Wellness (FFW) online behavioral intervention to increase well-being and physical activity in adults with obesity in the United States of America (USA). The FFW effectiveness trial described in this paper builds upon the results from a 2015 FFW efficacy trial [1]. Before describing either the 2015 FFW efficacy trial or the protocol for the current FFW effectiveness trial, however, we begin with a brief review of the population (i.e., adults with obesity) targeted in, and a primary outcome (i.e., promotion of physical activity) targeted by, the current study.

### Obesity

The World Health Organization (WHO) estimates that there are 650 million adults with obesity and that the number of adults with obesity has tripled since 1975 [2]. Obesity is a risk factor for major non-communicable diseases such as cardiovascular disease, type II diabetes, musculoskeletal disorders, and some cancers [3]. To reduce the prevalence of adults with obesity the WHO recommends that individuals limit energy intake from low quality food (e.g., highly processed foods high in fat), increase energy intake from high quality food (e.g., raw vegetables), and engage in regular physical activity (e.g., 150 min at moderate intensity per week). There is evidence, however, that very few (e.g., < 5%) adults with obesity meet public health guidelines for physical activity [4]. Fortunately, there also is evidence that well-designed cognitive-behavioral interventions can successfully promote physical activity in adults with obesity [5].

### Physical activity

Insufficient physical activity in the general adult population is a global pandemic [6, 7]. Successfully addressing this pandemic will require ongoing and wide implementation of a variety of intervention strategies (e.g., community-wide, informational, behavioral, social, policy, and built environment) at multiple levels of society (e.g., individual, neighborhood, municipality, country, etc.) across the globe [8, 9]. At the individual-level, there is evidence that behavioral interventions designed to promote physical activity by focusing on personal psychological attributes (e.g., self-efficacy) can be effective [10, 11]. Delivering a physical activity intervention online has been shown to be an effective mode of delivery [12] that also may allow for efficient scaling up of an intervention [9]. Thus, a readily scalable online behavioral intervention that effectively promotes physical activity in adults with obesity may be useful in regard to responding to a global pandemic (i.e., physical inactivity) in an at-risk population (i.e., adults with obesity).

### A conceptual summary of the FFW intervention

The conceptual framework for the FFW intervention is based on self-efficacy theory [13]. Self-efficacy theory resides within the more general social cognitive theory [14]. In social cognitive theory, individuals are regarded as proactive agents in the regulation of their cognition, motivation, actions and emotions. Self-efficacy judgments occupy a central role in self-efficacy theory and are defined as domain-specific beliefs held by individuals about their ability to successfully execute differing levels of performance given certain situational demands [13]. The formation of self-efficacy beliefs is believed to rely upon the cognitive processing of diverse sources of efficacy information that can be categorized as follows: past performance accomplishments, vicarious experiences, verbal persuasion, and physiological and/or emotional states [13]. Two proposed omnibus outcomes of self-efficacy beliefs are an individual’s thought patterns (e.g., goal setting; worry; and attributions) and behavior (e.g., challenges undertaken; effort expended on challenges undertaken; and persistence in the face of difficulties that arise during challenges undertaken). Over the past few decades, self-efficacy theory has been one of the most widely studied conceptual frameworks in exercise psychology [15].

### Fun for wellness

**Fig. 1:**
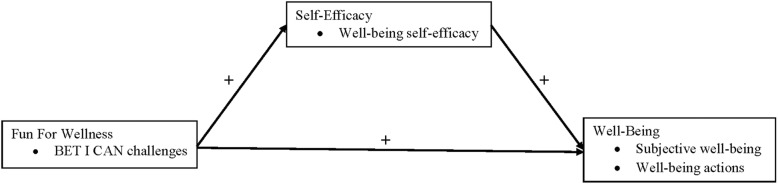
The conceptual model that guided the 2015 Fun For Wellness efficacy trial [1]

Fun For Wellness is an online behavioral intervention developed to encourage growth in well-being by providing capability-enhancing learning opportunities to participants [1]. The target audience of the FFW intervention is the adult population (e.g., adults with obesity) who are willing and able to engage with the online environment within which the intervention is delivered to participants. Figure 1 depicts the conceptual model that guided the 2015 FFW efficacy trial [1]. The FFW intervention (i.e., engagement with the BET I CAN challenges) was conceptualized as exerting both a positive direct effect, and a positive indirect effect through well-being self-efficacy, on both subjective well-being and well-being actions. Empirical evidence regarding the model in Fig. 1 will be reviewed after a conceptual summary of each component within Fig. 1 is provided.

#### BET I CAN challenges

Self-efficacy theory [13] provided the conceptual model that guided the creation of capability-enhancing learning opportunities (i.e., challenges) for participants to engage with in the FFW intervention. The capability-enhancing learning opportunities made available to participants are 152 interactive and scenario-based challenges that are ordered in the FFW website by the BET I CAN abbreviation [1]. The *B*ehavior-intensive learning opportunities focus on enhancing a person’s capabilities for both effective goal setting and forming positive habits [16]. The *E*motion-intensive learning opportunities focus on enhancing a person’s capabilities for both coping with negative feelings and collecting positive feelings [17]. The *T*hought-intensive learning opportunities focus on enhancing a person’s capabilities for both challenging negative expectations and creating a new life story [18]. The *I*nteraction-intensive learning opportunities focus on enhancing a person’s capabilities for both communicating and connecting with other people [19]. The *C*ontext-intensive learning opportunities focus on enhancing a person’s capabilities for both reading and changing cues in the environment [20]. The *A*wareness-intensive learning opportunities focus on enhancing a person’s capabilities for both knowing herself/himself and knowing the issue [21]. The *N*ext steps-intensive learning opportunities focus on enhancing a person’s capabilities for both making and sticking with a strategy [22]. To summarize, each BET I CAN challenge available in the FFW intervention was designed to provide an opportunity for an individual to increase their capabilities to organize and execute actions that may increase their well-being.

Every BET I CAN challenge in the FFW online behavioral intervention was designed in a way to expose an individual to one or more proposed sources of self-efficacy beliefs: enactive mastery experiences; vicarious experiences; verbal persuasion; and self-assessments of physiological and/or emotional states [13]. An example of exposure to an enactive mastery experience in the FFW online behavioral intervention is requiring an individual to engage with an interactive game to complete a BET I CAN challenge [1]. An example of exposure to a vicarious experience in the FFW online behavioral intervention is requiring an individual to watch a vignette of professional actors (some of whom may be classified as overweight and/or obese) modeling healthful behaviors to complete a BET I CAN challenge [1]. An example of exposure to verbal persuasion in the FFW online behavioral intervention is requiring an individual to listen to a coach deliver an animated lecture on healthful behaviors to complete a BET I CAN challenge [1]. An example of exposure to self-assessments of physiological and/or emotional states in the FFW online behavioral intervention is requiring an individual to engage in a self-reflection exercise to complete a BET I CAN challenge [1]. In summary, each of these capability enhancing-learning opportunities was guided by the substantial extant literature on the potential importance of proposed sources of self-efficacy information in exercise psychology [15, 23].

#### Self-efficacy

A necessary condition for valid testing of self-efficacy theory is a high degree of concordance between the domain specific self-efficacy beliefs and the proposed outcomes of the domain specific self-efficacy beliefs of interest [13]. The importance of maximizing concordance between efficacy beliefs and proposed outcomes of efficacy beliefs has been demonstrated in exercise psychology [24]. Bandura [25] advocated for the construction of study-specific self-efficacy scales as a mechanism for maximizing concordance between the domain specific self-efficacy beliefs and the proposed outcomes of the domain specific self-efficacy beliefs of interest. The conceptualization of self-efficacy beliefs within the FFW intervention was concordant with a multidimensional conceptualization of well-being [26].

##### Well-being self-efficacy

The operational definition for the well-being self-efficacy construct within the FFW conceptual framework is the degree to which an individual perceives that they have the capability to attain a positive status in key domains of their life [26]. As depicted in Fig. 1, exposure to the BET I CAN challenges was conceptualized as a positive predictor of well-being self-efficacy in the 2015 FFW efficacy trial. The seven specific dimensions of well-being self-efficacy targeted in the FFW online behavioral intervention are *I*nterpersonal (i.e., degree to which an individual perceives that they have the capability to attain well-being in…their relations with significant individuals), *C*ommunity (i.e., …the surrounding area within which they live), *O*ccupational (i.e., …their primary occupation), *P*hysical (i.e., …their wellness and physical health), *P*sychological (i.e., …their emotional experiences), *E*conomic (i.e., …their financial outlook) and overall (i.e., …the general status across the aforementioned areas of their life). The well-being self-efficacy (WBSE) scale [26] was piloted in the 2015 FFW efficacy trial [1] and was designed to measure only the overall dimension. Given that the FFW online behavioral intervention targets six dimensions of well-being self-efficacy (i.e., interpersonal, community, occupational, physical, psychological and economic) in addition to the overall dimension an expanded version of the WBSE scale was advocated for [26].

#### Well-being

As depicted in Fig. 1 the FFW intervention was hypothesized to exert both a positive direct effect, and a positive indirect effect through well-being self-efficacy, on well-being (i.e., subjective well-being and well-being actions). Within the FFW conceptual framework the well-being construct is based on both a multidimensional model of subjective well-being (e.g., how satisfied are you with your current wellness and physical health) and a multidimensional model of well-being actions (e.g., how many days per week do you engage in moderate physical activity for at least 30 min). From this point forward we generally use the succinct expression, well-being, to simultaneously refer to both subjective well-being and well-being actions as conceptualized within the FFW intervention.

##### Subjective well-being

The operational definition for the subjective well-being construct within the FFW conceptual framework is an individual’s satisfaction with their status in key domains of their life [27]. Within self-efficacy theory, subjective well-being can be viewed as residing within the omnibus outcome category of an individual’s thought patterns. The seven specific dimensions of subjective well-being targeted in the FFW intervention are *I*nterpersonal (i.e., how satisfied an individual is with… their relations with significant individuals), *C*ommunity (i.e., … the surrounding area within which they live), *O*ccupational (i.e., …their primary occupation), *P*hysical (i.e., …their wellness and physical health), *P*sychological (i.e., …their emotional experiences), *E*conomic (i.e., …their financial outlook) and overall (i.e., …the general status across the aforementioned key domains in their life). The I COPPE Scale [27] was designed to measure subjective well-being as conceptualized in the FFW intervention. The conceptual framework from which the I COPPE scale was developed was based on a broad consensus that well-being entails satisfaction with life as a whole and with specific sub-domains of well-being [28–30].

##### Well-being actions

The operational definition for the well-being actions construct within the FFW conceptual framework is as an individual’s actions that may improve their status in key domains of their life [31]. Within self-efficacy theory, well-being actions can be viewed as residing within the omnibus outcome category of an individual’s behavior. The six specific dimensions of well-being actions targeted in the FFW online behavioral intervention are *I*nterpersonal (i.e., an individual’s actions that may improve… their relations with significant individuals), *C*ommunity (i.e., …the surrounding area within which they live), *O*ccupational (i.e., …their performance in their primary occupation), *P*hysical (i.e., …their wellness and physical health), *P*sychological (i.e., …their emotional experiences), and *E*conomic (i.e., …their financial outlook). The I COPPE actions scale [32] was piloted in the 2015 FFW efficacy trial [1] and was designed to measure well-being actions. The conceptual framework from which the I COPPE actions scale was developed was very similar to the conceptual framework from which the I COPPE scale was developed. There is evidence that each of the dimensions of well-being targeted in the FFW intervention, except for economic, is applicable to exercise science [33].

### Results from the 2015 FFW efficacy trial

The purpose of the 2015 FFW efficacy trial was to deliver the first investigation of the efficacy of the FFW online behavioral intervention to increase well-being [1]. There were three key sets of findings from the 2015 FFW efficacy trial relevant to the current study. First, participants who complied with the FFW intervention had significantly higher subjective well-being scores, as compared to potential compliers in the UC group, in the following dimensions: interpersonal at 60 days (*p* = .042, Cohen’s *d* = 0.80), community at 30 days (*p* = .019, *d* = 0.71) and at 60 days (*p* = .046, *d* = 0.59), psychological at 60 days (*p* = .009, *d* = 0.56) and economic at 30 days (*p* = .007, *d* = 0.85) and at 60 days (*p* < .001, *d* = 0.94) post-baseline [1]. Second, the adjusted mean difference in overall well-being self-efficacy scores for participants who complied with the intervention, as compared to potential compliers in the UC group, was equal to 0.21, *p* = .061, *d* = 0.36 at 30 days and 0.28, *p* = .050, *d* = 0.49 at 60 days post-baseline [26]. Third, participants who complied with the FFW intervention, had significantly higher well-being actions scores, as compared to potential compliers in the UC group, in the interpersonal dimension at 60 days (*p* = .003, *d* = 0.78) and the physical dimension at 30 days (*p* = .044, *d* = 0.21) post-baseline [31]. Readers are referred to the main outcomes publication for a description of the methodological details in the 2015 FFW efficacy trial [1].

### A FFW effectiveness trial

**Fig. 2:**
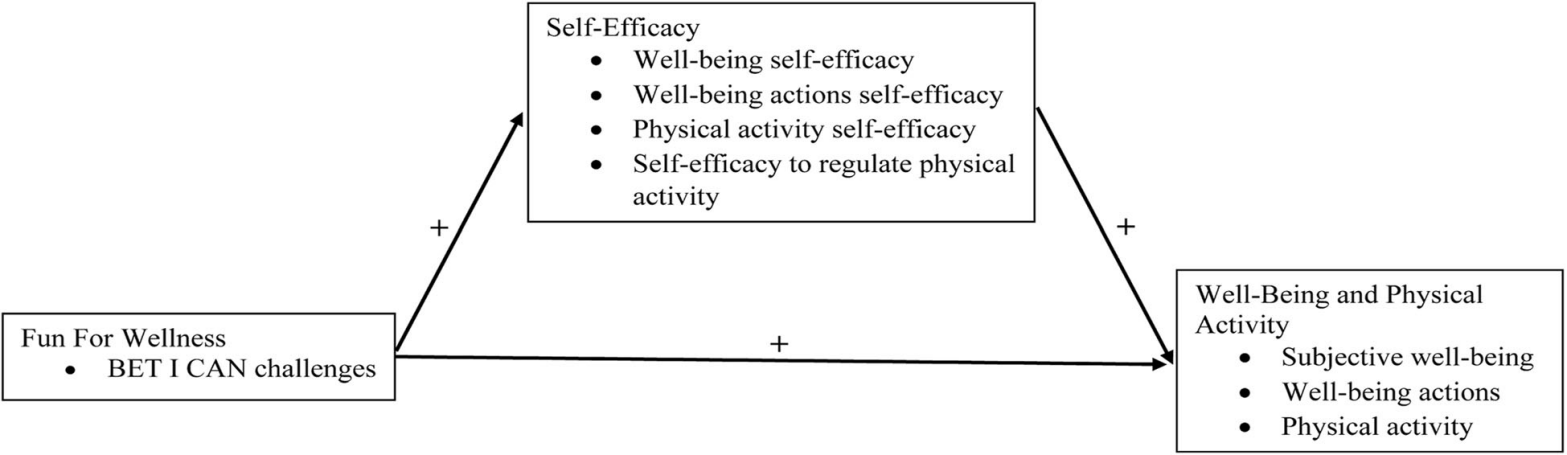
The conceptual model that will guide the for the Fun For Wellness effectiveness trial

The current study is designed to provide the first investigation of the effectiveness of the FFW online behavioral intervention to increase well-being and physical activity in adults with obesity in the USA. Figure 2 depicts the conceptual model that will guide the FFW effectiveness trial. The FFW online behavioral intervention (i.e., engagement with the BET I CAN challenges) is conceptualized as exerting both a positive direct effect, and a positive indirect effect through self-efficacy (i.e., well-being self-efficacy, well-being action self-efficacy, physical activity self-efficacy, self-efficacy to regulate physical activity), on well-being (i.e., subjective well-being, well-being actions, and physical activity).

### Additional constructs

Four constructs – well-being actions self-efficacy, physical activity self-efficacy, self-efficacy to regulate physical activity and physical activity – were added within the general framework of the original conceptual model (compare Fig. 1 to Fig. 2) in this effectiveness trial. The rationale for expanding the original conceptual model to include each of these constructs is provided below. Details for the measurement of each of these constructs in this study, however, will be discussed in the Method section.

#### Well-being actions self-efficacy

The conceptual model was expanded to include well-being actions self-efficacy for two primary reasons. The first reason was based on results from previous research where some evidence was provided for the efficacy of the FFW online behavioral intervention to increase well-being self-efficacy in those who complied with the intervention [26]. We reason that if exposure to the BET I CAN challenges can increase an individual’s self-efficacy regarding his or her well-being related thoughts (i.e., subjective well-being) then exposure to the BET I CAN challenges may also increase an individual’s self-efficacy regarding his or her well-being related behavior (i.e., well-being actions). The second reason for adding well-being actions self-efficacy to the current study was to maximize concordance between well-being actions and the measured set of self-efficacy beliefs. Recall that within the FFW conceptual framework the operational definition of the well-being self-efficacy construct (i.e., the degree to which an individual perceives that they have the capability to attain a positive status in key domains of their life) is more concordant with the operational definition of the subjective well-being construct (i.e., an individual’s satisfaction with their status in key domains of their life) than with the operational definition of the well-being actions construct (i.e., an individual’s actions that may improve their status in key domains of their life). The operational definition for the well-being actions self-efficacy construct in the FFW effectiveness trial is the degree to which an individual believes that they have the capability to take actions that may improve their status in key domains of their life.

#### Physical activity

The conceptual model was expanded to include physical activity for three primary reasons. The first reason was based on previous research where some evidence was provided for the efficacy of the FFW online behavioral intervention to increase physical well-being actions [31]. Measurement of physical well-being actions in the aforementioned study, however, consisted of only two items (e.g., how often do you engage in moderate physical activity such as brisk walking for about 30 min at least five times a week?). The current study seeks to more thoroughly measure physical activity. The second reason for adding physical activity to the current study was in response to calls for effective and scalable interventions to combat the global pandemic of physical inactivity [6, 7]. The final reason for adding physical activity to the current study was existing evidence that well-designed cognitive-behavioral interventions can successfully promote physical activity in adults with obesity [5]. The operational definition for physical activity in the FFW effectiveness trial is bodily movement produced by skeletal muscles that requires energy expenditure [34].

#### Physical activity self-efficacy

The conceptual model was expanded to include physical activity self-efficacy for three primary reasons. The first reason was based on results from previous research where some evidence was provided for the efficacy of the FFW online behavioral intervention to increase well-being self-efficacy beliefs [26] and physical well-being actions [31] in those who complied with the intervention. The second reason for adding physical activity self-efficacy to the current study was to maximize concordance between physical activity and the measured set of self-efficacy beliefs. The final reason for adding physical activity self-efficacy to the current study was existing evidence of a positive relationship between self-efficacy and physical activity in adults [10]. The operational definition for physical activity self-efficacy in the FFW effectiveness trial is the degree to which an individual perceives that they have the capability to engage in a recommended amount of weekly physical activity for health.

#### Self-efficacy to regulate physical activity

The conceptual model was expanded to include self-efficacy to regulate physical activity for reasons that closely follow the rationale already provided for including physical activity self-efficacy. The reason, however, for including both a self-efficacy “level” construct (i.e., physical activity self-efficacy) and a self-regulatory efficacy construct (i.e., self-efficacy to regulate physical activity) is that the former focuses on an individual’s beliefs in his or her ability to accomplish levels of a task while the latter focuses on an individual’s beliefs to overcome possible barriers to accomplishing a task that he or she already knows how to do [25]. Self-efficacy theory [13] posits that a self-efficacy level construct may play a central role in the initiation of a behavior (e.g., engaging in a recommended amount of weekly physical activity in week 1) while a self-regulatory efficacy may play a central role in the maintenance of a behavior (e.g., engaging in a recommended amount of weekly physical activity for six consecutive months). The importance of both a self-efficacy level construct and a self-regulatory efficacy construct has been demonstrated in exercise contexts [35, 36]. The operational definition for self-efficacy to regulate physical activity in the FFW effectiveness trial is the degree to which an individual perceives that they have the capability to overcome possible barriers to engagement in a recommended amount of weekly physical activity for health.

### Primary objective, outcomes, and hypotheses

The primary objective of the FFW effectiveness trial is to provide the first investigation of the effectiveness of FFW to increase well-being and physical activity in adults with obesity in the USA. Primary outcomes in the FFW effectiveness trial are: well-being self-efficacy, subjective well-being and well-being actions. These three constructs were chosen as primary outcomes based on some results from the 2015 FFW efficacy trial and the availability of at least some validity evidence for the relevant instrumentation (WBSE, I COPPE and I COPPE actions scales) prior to data collection. Three construct-level a priori hypotheses are listed below.

1. The FFW intervention will exert a positive direct effect on well-being self-efficacy.
2. The FFW intervention will exert a positive direct effect on subjective well-being.
3. The FFW intervention will exert a positive direct effect on well-being actions.

One construct-level exploratory hypothesis also will be investigated based on the conceptual model depicted in Fig. 2 and consistent with suggestions for future research on the FFW intervention [1, 26].

1.The FFW intervention will exert a positive indirect effect on subjective well-being through well-being self-efficacy

Dimension-specific hypotheses for primary outcomes were not made due to a lack of previous research on the effectiveness of the FFW intervention.

### Secondary outcomes and hypotheses

Secondary outcomes in the FFW effectiveness trial are: well-being actions self-efficacy, physical activity self-efficacy, self-efficacy to regulate physical activity, and physical activity. These four constructs were chosen as secondary outcomes because validity evidence for the relevant instrumentation was not available prior to data collection and/or because they were not included in the 2015 FFW efficacy trial. Four construct-level a priori hypotheses with the intervention are listed below.

1. The FFW intervention will exert a positive direct effect on well-being actions self-efficacy.
2. The FFW intervention will exert a positive direct effect on physical activity self-efficacy.
3. The FFW intervention will exert a positive direct effect on self-efficacy to regulate physical activity.
4. The FFW intervention will exert a positive direct effect on physical activity.

Two construct-level exploratory hypotheses also will be investigated based on the conceptual model depicted in Fig. 2 and consistent with suggestions for future research on the FFW intervention [31].

1. The FFW intervention will exert a positive indirect effect on well-being actions through well-being actions self-efficacy.
2. The FFW intervention will exert a positive indirect effect on physical activity through physical activity self-efficacy and self-efficacy to regulate physical activity.

Dimension-specific hypotheses for secondary outcomes were not made due to a lack of previous research on the effectiveness of the FFW intervention.

## Methods/design

### Ethical approval

Each procedure in the current study that involves one or more human participants will be in accord with both the 1964 Helsinki declaration and its later amendments or comparable ethical standards and with the ethical standards of the institutional and/or national research committee. The University of Miami’s institutional review board provided needed authorization to conduct this study on 11 July 2017, IRB# 20170541. Table 1 provides the Standard Protocol Items: Recommendations for Interventional Trials (SPIRIT) flow diagram. A populated SPIRIT checklist is provided in the Appendix within Additional file 1.

**Table 1:** SPIRIT flow diagram for the FFW effectiveness trial

	STUDY PERIOD					
	Enrolment	Allocation	Post-allocation			Close-out
TIMEPOINT**	***-t***_***1***_	**0**	***t***_***1***_ ***day 0***	***t***_***2***_ ***day 30***	***t***_***3***_ ***day 60***	***t***_***x***_
ENROLMENT:						
Eligibility screen	X					
Informed consent	X					
Allocation		X				
INTERVENTIONS:						
*Fun For Wellness*			X	X		
*Usual Care*			X	X	X	
ASSESSMENTS:						
*Gender*			X			
*Age*			X			
*Race*			X			
*Education-level*			X			
*Marital status*			X			
*Annual income*			X			
*Zip code*			X			
*Height*			X	X	X	
*Weight*			X	X	X	
*Self-efficacy to comply*			X			
*Well-being self-efficacy*			X	X	X	
*Well-being actions self-efficacy*			X	X	X	
*Physical activity self-efficacy*			X	X	X	
*Self-efficacy to regulate physical activity*			X	X	X	
*Subjective well-being*			X	X	X	
*Well-being actions*			X	X	X	
*Physical activity*			X	X	X	
*Fun For Wellness Experience Survey*				X		

### Informed consent

Each eligible individual who wishes to be a participant in the study will be required to provide informed consent. More precisely, immediately after being determined to be eligible for this study, each eligible individual will be directed to a web-based IRB-approved informed consent form. Each individual who clicks the “Consent to Participate” will be enrolled as a participant in the study. Each individual who clicks “Decline to Consent” will be denied access to the intervention.

### Study design

The study design is a large-scale, prospective, parallel group RCT. Recruiting, verification of eligibility, random assignment and collection of data will be conducted online. Data collection will occur at baseline (Time 1), 30 days (Time 2) and 60 days (Time 3) after baseline. The timeline for this study is similar to timelines used in other well-being [37] and physical activity interventions [5]. The probability of rejecting a truly false null hypothesis (i.e., statistical power) for every focal parameter was estimated a priori and each of these estimates will be provided in the Data Analytic Approach section.

#### Recruitment

Approximately 9 hundred participants will be enrolled in this study. Participants will be recruited through the SurveyHealth (http://www.surveyhealthcare.com/) company’s panel recruitment based on the eligibility criteria for this study. Partnering with a panel recruitment company is consistent with recruitment in previous research on FFW [26, 27] and with a movement toward larger and smarter approaches that promote physical activity [9].

#### Eligibility

There are five eligibility criteria for participation in this study. The first eligibility criterion is the ability to access the online intervention. This criterion will be assessed by asking each participant to confirm that he or she will have access to a technological device (e.g., smartphone) that can access the online intervention via a web browser throughout the study. The second eligibility criterion is living in the USA. This criterion will be assessed by asking each participant to confirm that he or she is living in the USA. A justification for this criterion is evidence for differences in the prevalence of physical activity in adults by country [7]. The third eligibility criterion is being an adult between 18 and 64 years old. This criterion will be assessed by asking each participant the year in which he or she was born. A justification for this criterion is that our target population is adults (i.e., 18–64 years) and not older adults (i.e., 65 years and above) based on evidence-based age groupings for global recommendations on physical activity for health [38]. The fourth eligibility criterion is a body mass index (BMI) ≥ 25.00 kg/m^2^. Our BMI criterion includes the overweight category (i.e., 25.00–29.99 kg/m^2^) as well as the obese category (i.e., ≥ 30.00 kg/m^2^) consistent with many interventions promoting physical activity in obese populations [5]. This criterion will be assessed by asking each participant his or her height and weight. A justification for this criterion is trying to promote physical activity in a population where very few individuals may meet public health guidelines for physical activity [4]. The final eligibility criterion is the absence of simultaneous enrollment in another intervention program promoting either well-being or physical activity. This criterion will be assessed by requiring each participant to confirm that he or she will not be enrolled in another formal intervention program promoting either well-being or physical activity during the FFW study period. A justification for this criterion is a reduction in the likelihood of confounding the results from the current study with results that may be due to enrollment in other programs. The short form of the international physical activity questionnaire (IPAQ; [39, 40]) and the physical activity readiness questionnaire [41] will also be included in the screener and responses to these instruments may be used for exploratory purposes.

#### Random assignment

Eligible participants will be randomly assigned to the intervention (i.e., FFW) or the wait-list control (i.e., UC) group via software code that is written to accomplish equal (i.e., balanced) allocations to the FFW and UC groups. Research staff will be blinded to participant group assignment. The outcomes assessor will be blinded to participant group assignment.

##### Usual care

Participants assigned to the UC group will be prompted to use the unique and secure log-in credentials they will be provided when they are screened for the study. These participants will be asked to conduct their lives as usual during the intervention period. The unique and secure log-in credentials will, however, provide them with access to a secure website to complete the Time 1 battery, Time 2 battery, and the Time 3 battery. Participants allocated to the UC group will have an opportunity to receive up to $30 worth of Amazon electronic gift cards. Specifically, at Time 1 they may receive $5 (for completing the Time 1 battery), $10 at Time 2 (for completing the Time 2 battery), and $15 at Time 3 (for completing the Time 3 battery). Participants in the UC group also will be given 1 month of 24 h access to the 152 BET I CAN challenges after data collection for the effectiveness trial is closed.

##### Fun for wellness

Participants allocated to the FFW group will be prompted to use the unique and secure log-in credentials they will be provided when they are screened for the study. These credentials will provide them with 30 days (i.e., from Time 1 to Time 2) of 24 h access to the 152 BET I CAN challenges, as well as access to a secure website to complete the Time 1 battery, Time 2 battery, and the Time 3 battery. These participants will have the opportunity to receive a total of $45 worth of Amazon electronic gift cards. Specifically, at Time 1 they may receive $5 (for completing the Time 1 battery), $10 at Time 2 (for completing both the Time 2 battery and 15 BET I CAN post-introductory challenges) plus $15 more at Time 2 (for completing both the Time 2 battery and 30 BET I CAN post-introductory challenges), and $15 at Time 3 (for completing the Time 3 battery). The remuneration plan at Time 2 is linked to completing post-introductory challenges in an effort to increase engagement with the intervention. In the 2015 FFW efficacy trial the remuneration plan at Time 2 for FFW participants was not linked to completing post-introductory challenges.

There are four introductory challenges that focus on orienting participants to the FFW online behavioral intervention (e.g., an overview of the organization of the website; a brief introduction to the characters that perform in the vignettes; etc.). Participants must complete these introductory challenges to be able to access to the subsequent 148 non-introductory challenges. The non-introductory challenges are ordered in the FFW website by the BET I CAN abbreviation. Participants will self-select which non-introductory challenges to engage with. Challenges engaged with by each participant will be logged by computer software to provide information for the FFW engagement scoring system. This logging of challenges engaged with by each participant will be possible because access to the FFW online behavioral intervention will require each individual to use their unique and secure log-in credentials.

The operational definition for engagement (or equivalently, compliance) with the FFW online behavioral intervention will adhere to the FFW engagement scoring system described in the main outcomes paper for the 2015 FFW efficacy trial [1]. Within the FFW engagement scoring system, the potential impact of completing a given non-introductory challenge is initially classified as low impact (7 participation points), moderate impact (14 participation points) or high impact (21 participation points). The operational definition of full participation will be guided by both methodological (e.g., the identification of a sufficient number of compliers) and substantive (e.g., an individual would have to engage with the FFW online behavioral intervention for approximately 2 h to earn enough participation points) concerns as recommended in the compliance literature [42]. The operational definition for full participation is earning at least 21 participation points.

#### Data collection

Instruments to measure demographic information, anthropometric characteristics, self-efficacy, well-being and physical activity will be included in the battery. Data on literature-based demographic covariates of multidimensional well-being [43] will be collected at Time 1 and will include participant gender, age, race, education-level, marital status, and annual income. Zip code data will be collected as a proxy for a host of built environment factors that may be related to an individual’s level of physical activity [10]. Anthropometric data will be collected at all three time points and will be assessed by asking each participant his or her height and weight. Demographic, zip code, and anthropometric variables are collectively referred to as covariates from this point forward.

##### Self-efficacy to comply

Following completion of the four introductory challenges, each participant assigned to the FFW group will be asked to respond to the following item: *How confident are you in your current ability to get yourself to complete at least 15 Fun For Wellness post-introductory challenges within the next 30 days?* A five category rating scale structure, where 0 = no confidence, 1 = low confidence, 2 = moderate confidence, 3 = high confidence and 4 = complete confidence, will be implemented for this item, and in all self-efficacy scales from this point forward, based on previous research on effective self-efficacy rating scale structures [44]. Asking a participant at the onset of an intervention to estimate their potential engagement with the forthcoming intervention is consistent with some related previous research on compliance [45].

##### Well-being self-efficacy

Well-being self-efficacy will be measured at Time 1 through Time 3 with an expanded version (i.e., from 7-items to 21-items) of the WBSE scale as recommended in the literature [26]. The expanded version of the WBSE scale is highly concordant with subjective well-being as conceptualized in the FFW context (i.e., evaluated with the I COPPE scale). Specifically, the seven dimensions of well-being self-efficacy purported to be measured by the WBSE scale – interpersonal, community, occupational, physical, psychological, economic and overall – match the seven dimensions of subjective well-being purported to be measured by the I COPPE scale. Each of the seven dimensions of well-being self-efficacy purported to be measured by the WBSE scale has an exclusive item stem that references three unique periods of time: past (i.e., 30 days ago), present (i.e., right now), and future (i.e., 30 days from now). Reference to the past, present, and future is an established practice in the assessment of subjective well-being related constructs in general [46, 47] and in the FFW context [27, 48]. Estimates of the validity and reliability of scores resulting from answers to the original WBSE scale have been provided [26]. Estimates of the validity and reliability of scores resulting from answers to the expanded version of the WBSE scale, however, will not be available prior to data collection at Time 1. Evidence for the reliability and validity of responses to the expanded version of the WBSE scale will be evaluated prior to testing either the primary or secondary hypotheses.

##### Well-being actions self-efficacy

Well-being actions self-efficacy will be measured at Time 1 through Time 3 with the well-being actions self-efficacy (WBASE) scale, which is a newly developed 18-item instrument. The WBASE scale was designed to measure well-being actions self-efficacy as conceptualized in the FFW intervention. The WBASE scale is highly concordant with well-being actions as conceptualized in the FFW context (i.e., evaluated with the I COPPE actions scale). Specifically, the six dimensions of well-being actions self-efficacy purported to be measured by the WBASE scale – interpersonal, community, occupational, physical, psychological, and economic – match the six dimensions of well-being actions purported to be measured by the I COPPE actions scale. Each of the six dimensions of well-being actions self-efficacy purported to be measured by the WBASE scale has three items designed to measure it. Estimates of the validity and reliability of scores resulting from answers to the WBASE scale will not be available prior to data collection at Time 1. Evidence for the reliability and validity of responses to the WBASE scale, however, will be evaluated prior to testing either the primary or secondary hypotheses.

##### Physical activity self-efficacy

Physical activity self-efficacy will be measured at Time 1 through Time 3 with the physical activity self-efficacy (PASE) scale, which is a slightly modified version of the 8-item exercise self-efficacy (EXSE; [35]) scale. The EXSE scale assesses an individual’s beliefs in their ability to continue exercising on a three time per week basis at moderate intensities for 40+ minutes per session in the future. The PASE scale was tailored for the FFW context to assess the degree to which an individual perceives that they have the capability to engage in a recommended amount of weekly physical activity for health. The PASE scale is highly concordant with physical activity as conceptualized in the FFW conceptual model (i.e., measured by the IPAQ) in that it measures weekly physical activity across four general domains of life: leisure time (12-items), domestic and gardening (12-items), work-related (12-items), and transport-related (12-items). Each of the four domains has two unique stems (e.g., how confident are you in your current ability to engage in leisure related physical activity at a vigorous level of intensity) that reference six increasing time periods (e.g., for at least 10 or 15 or 30 or 45 or 60 or 75 min in the next week). Estimates of the validity and reliability of scores resulting from answers to the PASE scale will not be available prior to data collection at Time 1. Evidence for the reliability and validity of responses to the PASE scale, however, will be evaluated prior to testing either the primary or secondary hypotheses.

##### Self-efficacy to regulate physical activity

Self-efficacy to regulate physical activity will be measured at Time 1 through Time 3 with the self-efficacy to regulate physical activity (SERPA) scale, which is a slightly modified version of the 13-item barriers self-efficacy (BARSE; [36]) scale. The BARSE scale assesses an individual’s perceived capabilities to exercise three times per week for 40 min over the next 2 months in the face of commonly identified barriers to participation. The SERPA scale was tailored for the FFW context to assess the degree to which an individual perceives that they have the capability to overcome possible barriers to engagement in a recommended amount of weekly physical activity for health. The SERPA scale is highly concordant with physical activity as conceptualized in the FFW conceptual model (i.e., measured by the IPAQ). Estimates of the validity and reliability of scores resulting from answers to the SERPA scale will not be available prior to data collection at Time 1. Evidence for the reliability and validity of responses to the revised SERPA scale, however, will be evaluated prior to testing either the primary or secondary hypotheses.

##### Subjective well-being

Subjective well-being will be measured at Time 1 through Time 3 with the 21-item I COPPE scale, which is available in Prilleltensky et al. [27]. Each of the seven dimensions of subjective well-being purported to be assessed by the I COPPE scale – interpersonal, community, occupational, physical, psychological, economic and overall – is measured with an exclusive item stem that references three unique periods of time: past (i.e., 30 days ago), present (i.e., right now), and future (i.e., 30 days from now). Responses to each item are organized within an 11-point Likert scale that ranges from 0 (*worst your life can be*) to 10 (*best your life can be*). Estimates of the validity and reliability of scores resulting from answers to the I COPPE scale have been provided [1, 27, 33, 48]. The Optum™ SF-36v2 Health Survey [49] will also be included in the battery for possible exploratory purposes.

##### Well-being actions

Well-being actions will be measured at Time 1 through Time 3 with an expanded version (i.e., from 12-items to 18-items) of the I COPPE actions scale [32]. The I COPPE actions scale was expanded from 12-items to 18-items to match the WBASE scale. Each of the six dimensions of well-being actions purported to be measured by the I COPPE actions scale – interpersonal, community, occupational, physical, psychological, and economic – has three items designed to measure it. Responses to each item are organized within a 7-point Likert scale that ranges from 0 (*never*) to 6 (*always*). Estimates of the validity and reliability of scores resulting from answers to the original 12-item I COPPE actions scale have been provided [31, 32]. Estimates of the validity and reliability of scores resulting from answers to the expanded version of the I COPPE actions scale, however, will not be available prior to data collection at Time 1. Evidence for the reliability and validity of responses to the expanded version of the I COPPE actions scale will be evaluated prior to testing either the primary or secondary hypotheses.

##### Physical activity

Physical activity will be measured at Time 1 through Time 3 with the long form (i.e., 27-items) of the IPAQ [39, 40]. The long form of the IPAQ is intended for individuals from 15 to 69 years old and purports to measure physical activity in four domains – leisure time, domestic and gardening, work-related, and transport-related – according to the frequency and duration of the physical activity performed in each domain during a week. The physical activities measured are separated according to their intensity, which is defined as a distinction between walking, moderate physical activities, and vigorous physical activities. Moderate activities are those that cause a small increase in respiratory frequency and require moderate physical exertion, and vigorous activities cause more breathing than normal, with hard physical exertion [40]. Physical activity scores will be created based on IPAQ data processing guidelines [50].

### Data analytic approach

Evidence for the reliability and validity of responses to each revised (e.g., WBSE scale) or new (e.g., WBASE scale) instrument will be evaluated prior to testing either the primary or secondary hypotheses consistent with standards for psychological testing [51]. Exploratory structural equation modelling will be used to fit Time 1 data [52]. Latent variable reliability will be measured with coefficient *H* [53]. Composite score reliability will be assessed with coefficient omega [54] and coefficient alpha [55].

To test the a priori hypotheses previously stated, three general models will be fit for each primary and secondary outcome in M*plus* 8.0 [56]. The estimator for each model will be maximum-likelihood with standard errors that are robust to conditional non-normality. In each model the main focus will be to examine the mean difference between the FFW group and the UC group on a proposed outcome at both Time 2 and at Time 3. Alternate specifications to each model will also be considered to examine some model-based assumptions [57]. The first model (i.e., Model A) will follow an intent-to-treat approach [58] by estimating the effect of being allocated to the treatment (i.e., FFW in this case) condition (i.e., ITT or γ). The second model (i.e., Model B) will follow a complier average causal effect (i.e., CACE) approach [59–62] by estimating the effect of being allocated to the treatment (i.e., FFW in this case) condition for compliers with the FFW intervention (i.e., γ_c_). The third model (i.e., Model C) will follow a CACE approach by estimating the effect of being allocated to the treatment (i.e., FFW in this case) condition for non-compliers with the FFW intervention (i.e., γ_nt_) in addition to estimating γ_c._ Fitting Model C provides a way of evaluating the sensitivity of Model B [45]. Model B and Model C both employ CACE estimation, where non-compliers will be conceptualized as never-takers consistent with CACE methodology based assumptions detailed in relevant literature [59–62]. If level of engagement is below 50% then a CACE approach (e.g., Model B and C) will be favored [63]. If level of engagement is at least 50% then an ITT approach (e.g., Model A) will be favored [63]. For exploratory hypotheses bias-corrected bootstrapped estimates of 95% confidence intervals for indirect effects within a path model will be obtained with the number of draws set equal to 2000 under an ITT approach [64]. Missing data (e.g., dropout) will be reported (e.g., in a flow diagram) and modeled consistent with the missing at random assumption [65] consistent with previous FFW research [1].

#### Type I error

The probability of falsely rejecting a true null hypothesis (i.e., α) will be set to equal .05. This approach is consistent with a majority of RCTs with multiple outcomes, where a downward adjustment to α generally has not been applied to maximize statistical power (in the event that a null hypothesis is false) [66]. To address, however, a reasonable concern with the possibility of an inflated α (in the event that a null hypothesis is true), when statistical significance is observed for a focal parameter we will emphasize an effect size estimate; provide a 95% confidence interval for the effect size estimate; and, explicitly note that caution should be exercised with regard to observed statistical significance until confirmatory studies become available in the future [66].

#### Effect size

Effect size will be estimated in each model by dividing the mean difference by the square root of the variance pooled across the UC and FFW groups. In Model A this effect size estimate is equivalent to Cohen’s *d* [67]. In Model B and Model C this effect size estimate can be regarded as an extension of Cohen’s *d* to a latent class framework [57]. In an effort to gain some textual parsimony, we will denote the estimated effect size in each model as Cohen’s *d* hereto forward. Similarly, we will use heuristics put forth by Cohen [67] to describe the magnitude of the absolute value of Cohen’s *d*: 0.20, (small), 0.50 (medium) and 0.80 (large).

#### Model A in more detail

Model A will impose a regression model for each proposed outcome with measures taken at Time 2 and Time 3 as the dependent variables. Covariates, the outcome at Time 1, and group allocation (FFW =1, UC = 0) will serve as predictors of the outcome at Time 2 and Time 3 and these regression coefficients will be estimated freely. Intercepts for the outcome at Time 2 and Time 3 will be estimated freely. Covariance between the error terms for the outcome at Time 2 and Time 3 will be estimated freely. The focal parameters will be the direct effects from group allocation to the outcome at Time 2 (γ_Time2_) and Time 3 (γ_Time3_). A positive focal parameter value will convey that the FFW group has a higher adjusted mean for the outcome as compared to the UC group.

#### Model B in more detail

Model B will impose a latent class (with two classes) regression model with CACE estimation for each proposed outcome with measures taken at Time 2 and Time 3 as the dependent variables. The first class (i.e., Class 1) will be conceptualized as never-takers. The second class (i.e., Class 2) will be conceptualized as compliers. A dichotomous indicator of latent class (where 0 = non-compliers in the FFW group, 1 = compliers in the FFW group, and a missing value for participants in the UC group) will be generated. Compliance classification, modeled as a categorical latent variable, will be regressed on covariates. Covariates, the outcome at Time 1 and group allocation will serve as predictors of the outcome at Time 2 and Time 3 and these regression coefficients will be freely estimated in Class 1 and Class 2. The two direct effects from group allocation to the outcome at Time 2 and Time 3 will be fixed to 0 in Class 1 (i.e., the exclusion restrictions: γ_ntTime2_ = γ_ntTime3_ = 0), and will be estimated freely in Class 2 (i.e., γ_cTime2_, γ_cTime3_). The intercepts for the outcome at Time 2 and Time 3 will be estimated freely in each class. Covariance between the error terms for the outcome at Time 2 and Time 3 will be estimated freely in each class. The focal parameters will be the direct effects from group allocation to the outcome at Time 2 and Time 3 in Class 2 (i.e., γ_cTime2_, γ_cTime3_).

A positive focal parameter value will convey that compliers in the FFW group had a higher adjusted mean for the outcome as compared to potential compliers in the UC group.

#### Model C in more detail

Model C will estimate each parameter estimated in Model B while relaxing the exclusion restriction (i.e., freely estimate γ_ntTime2_ and γ_ntTime3_), making Model B nested within Model C. The change in the likelihood ratio *χ*^2^ (robust) test $, \Delta {\chi}_R^2$_,_ will formally compare the fit of these nested models. There is a substantive and a methodological rationale for evaluating the plausibility of the exclusion restriction assumption in the FFW online behavioral intervention. From a substantive standpoint the researchers may expect, based on results from the 2015 FFW efficacy trial [1], that some of the participants allocated to the FFW group may engage with the intervention at a level that yields a FFW engagement score greater than 0 (i.e., no engagement) but less than 21 (i.e., full participation). From a methodological standpoint it is important to note that it is well-known that the estimate of γ_c_ can be biased when the true γ_nt_ effect is not zero but is forced to equal zero, particularly when compliance with the intervention is less than high [45]. Therefore, the focal parameters in this model will be both the γ_cTime2_ and γ_cTime3_ effects and the γ_ntTime2_ and γ_ntTime3_ effects.

#### Statistical power estimation

The probability of rejecting a truly false null hypothesis for every focal parameter (i.e., γ_cTime2_, γ_cTime3_) was estimated (*N* = 900) in M*plus* 8.0 using Monte Carlo methods [68] under the assumption that engagement is likely to be less than 50% [1]. For each of the focal parameters in Model B the population parameter value equaled a value that corresponded to either a small (i.e., *d* = 0.20), moderate (i.e., *d* = 0.50), or large (i.e., *d* = 0.80) positive effect. A range of effect sizes were modeled consistent with relevant recommendations in exercise science [69]. The population model assumed a engagement rate of 25, 45%, or 65% based upon results observed in the 2015 FFW efficacy trial [1]. In the 2015 FFW efficacy trial engagement ranged from 15.6 to 54.9% with a mean of 31.6% across dimensions of well-being [1]. Our simulations assume a ~ 10% increase in engagement, which we believe may result from the new remuneration plan. Missing data (i.e., 35% at Time 2 and 40% at Time 3) were modeled based upon results observed in the 2015 FFW efficacy trial [1]. The quantity of replications requested equaled 10,000. Each replication was originally drawn from a conditionally multivariate normal distribution.

Table 2 provides the power estimation for γ_cTime2_ and γ_cTime3_ at Time 2 and Time 3. Power estimation for a small effect ranged from .30 (25% engagement) to .74 (65% engagement). Power estimation for a moderate effect ranged from .95 (25% engagement) to 1.00 (at least 45% engagement). Power estimation for a large effect equaled 1.00. We conclude that we are likely to have low to moderate power for small effects (depending on engagement level) and high power for moderate and large effects. Budgetary constraints preclude enrollment of more than approximately 900 participants.

**Table 2:** Power Estimation for the Complier Average Causal Effect at Time 2 and at Time 3

Effect size	Engagement	Power estimation at Time 2	Power estimation at Time 3
0.20	25%	0.32	0.30
0.20	45%	0.54	0.51
0.20	65%	0.74	0.71
0.50	25%	0.96	0.95
0.50	45%	1.00	1.00
0.50	65%	1.00	1.00
0.80	25%	1.00	1.00
0.80	45%	1.00	1.00
0.80	65%	1.00	1.00

## Discussion

Fun For Wellness is an online behavioral intervention developed to encourage growth in well-being by providing capability-enhancing learning opportunities to participants [1]. The purpose of this study is to provide the first investigation of the effectiveness of the FFW online behavioral intervention to increase well-being and physical activity in adults with obesity in the USA. The effectiveness trial described in this paper builds upon the 2015 FFW efficacy trial [1] and has the potential to be important for at least three reasons. The first reason that this study has the potential to be important is based upon a general scientific approach that the potential utility of interventions should be evaluated under both ideal (e.g., more controlled) and real-world (e.g., less controlled) conditions [70]. We believe that the FFW effectiveness trial described in this manuscript can be viewed as occurring within a less controlled context (e.g., recruitment within the USA via a panel recruitment company) as compared to the context within which the 2015 FFW efficacy trial [1] occurred (e.g., recruitment within a major research university). The second reason that this study has the potential to be important is based upon the global need for readily scalable online behavioral interventions that effectively promote physical activity in adults [9]. We believe that FFW may have the potential to eventually become useful, perhaps in a small but important way given the magnitude of the problem, in regard to responding to the global pandemic of physical inactivity. The third reason that this study has the potential to be important is based upon the troubling global trend toward obesity along with evidence for obesity as a risk factor for several major non-communicable diseases [2]. We believe that FFW, because of its conceptual basis in self-efficacy theory, may be effective in promoting physical activity in adults with obesity [5].

We are aware of at least four noteworthy limitations for this study. First, we acknowledge that there is some ambiguity concerning the utility of our operational definition of engagement (i.e., full participation). Although the operational definition of engagement in the current study will be consistent with previous research [1], we encourage continuing efforts to further understand engagement with the FFW intervention. Second, we recognize that our hypotheses assume additivity of FFW effects for all demographic covariates (i.e., no a priori moderators for the proposed effects of FFW). We encourage future secondary analyses that explore the prospect of heterogeneous FFW effects for sub-groups of individuals on well-being and/or physical activity. Third, we note that another limitation is that all of the data collected, except for engagement, will be collected via self-report. Future studies that collect proposed outcome data (e.g., well-being actions and physical activity) from more objective methods are encouraged. A final limitation is that the sample may be drawn from a somewhat broad population. Because recruitment for the study will occur via a partnership with a panel recruitment company, it is possible that a very heterogeneous sample may be enrolled. Subsequent studies that sample from a more narrowly defined population (e.g., adults with obesity who also suffer from a particular non-communicable disease) may provide different results.

## Additional file

Additional file 1: SPIRIT 2013 Checklist: Recommended items to address in a clinical trial protocol and related documents (DOC 189 kb)

## Data Availability

Not applicable.

## Abbreviations

BARSE: Barriers self-efficacy
BET I CAN: Behaviors, emotions, thoughts, interaction, context, awareness next steps
BMI: Body mass index
CACE: Complier average causal effect
EXSE: Exercise self-efficacy
FFW: Fun for wellness
I COPPE: Interpersonal, community, occupational, physical, psychological, economic
IPAQ: International physical activity questionnaire
ITT: Intent to treat
MET: Metabolic equivalent
MVPA: Moderate to vigorous physical activity
PASE: Physical activity self-efficacy
RCT: Randomized controlled trial
SERPA: Self-efficacy to regulate physical activity
SPIRIT: Standard Protocol Items: Recommendations for Interventional Trials
Time 1: baseline
Time 2: 30-days after baseline
Time 3: 60-days after baseline
UC: Usual care
USA: United States of America
WBSE: Well-being self-efficacy
WHO: World Health Organization
γ: intent to treat effect
γ_c_: effect for compliers
γ_cTime2_: effect for compliers at Time 2
γ_cTime3_: effect for compliers at Time 3
γ_nt_: effect for never-takers
γ_ntTime2_: effect for never-takers at time 2
γ_ntTime3_: effect for never-takers at Time 3
γ_Time2_: intent to treat effect at Time 2
γ_Time3_: intent to treat effect at Time 3
